# Molecular Characterization of PARP Inhibitor Response Reveals Co-Targeting Strategies in Advanced Prostate Cancer

**DOI:** 10.3390/cancers18152381

**Published:** 2026-07-23

**Authors:** Bryan Correa Gonzalez, Akshaya Karthikeyan, Love A. Moore, Anamitra Bhaumik, Ethan Sandoval, Marion Hardy, Ryan R. Davis, Neelu Batra, Christopher A. Lucchesi, Allen C. Gao, Hong Li, John D. McPherson, Marc Dall’Era, Alan P. Lombard

**Affiliations:** 1 Department of Urologic Surgery, University of California, Davis, Sacramento, CA 95817, USA; 2 Department of Molecular and Cellular Biology, University of California, Davis, Davis, CA 95616, USA; 3 Comprehensive Cancer Center, University of California, Davis, Sacramento, CA 95817, USA; 4 Department of Pathology and Laboratory Medicine, University of California, Davis, Sacramento, CA 95817, USA; 5 VA Northern California Health Care System, Sacramento, CA 95655, USA; 6 Department of Public Health Sciences, University of California, Davis, Sacramento, CA 95817, USA; 7 Department of Biochemistry and Molecular Medicine, University of California, Davis, Sacramento, CA 95817, USA

**Keywords:** prostate cancer, poly (ADP-ribose) polymerase (PARP) inhibitor (PARPi), olaparib, talazoparib, ATM, lartesertib (M4076), AZD1390, epithelial–mesenchymal transition (EMT), fatty acid metabolism, etomoxir, fatostatin

## Abstract

**Background/Objectives**: Though PARP inhibition has improved the management of advanced prostate cancer, patient outcomes may be modest and disease progression on treatment is common. We sought to improve understanding of tumor cell response to PARP inhibition to support development of novel strategies to enhance and/or prolong PARP inhibitor (PARPi) efficacy. **Methods**: Cell viability assays and microscopy were used for initial characterization of PARPi response in models of advanced prostate cancer. RNA sequencing was performed to investigate time-dependent transcriptomic changes induced by PARP inhibition. Western blots, flow cytometry, and both additional viability assays and microscopy were used to validate RNA sequencing results and test potential therapeutic strategies. **Results**: Characterization of responses to PARP inhibition reveals time-dependent changes which may be targeted to improve treatment efficacy. In line with the expected PARPi mechanism of action, short-term treatment is largely associated with activation of ATM and the DNA damage response and cell cycle checkpoint signaling. Targeting ATM with clinical stage inhibitors significantly enhances reduction of tumor cell viability by PARP inhibition. Tumor cells exposed to longer-term treatment exhibit SLUG-dependent epithelial–mesenchymal transition (EMT) and evidence for altered fatty acid metabolism, both of which may be targeted to enhance PARPi anti-tumor cell effects. **Conclusions**: This study provides insight into both short and longer-term cellular response to PARPi treatment and provides a foundation for additional efforts to explore effective strategies to maximize the utility of PARP inhibition for managing prostate cancer.

## Simple Summary

Though clinically impactful, PARP inhibitor efficacy may be modest and disease progression on treatment is a frequent occurrence. We sought to improve characterization of tumor cell response to PARP inhibition to support efforts to design novel strategies aimed at either (1) exacerbating the direct effects of PARP inhibitors on tumor cells or (2) targeting adaptive mechanisms utilized by tumor cells to survive and progress on treatment. The presented data highlight the potential of using clinical stage inhibitors of ATM to increase PARP inhibitor effectiveness and suggest that PARP inhibitor-induced epithelial–mesenchymal transition and metabolic changes may represent targetable vulnerabilities. These findings provide a foundation on which to further explore development of new approaches for augmenting PARP inhibitor-based management of advanced prostate cancer.

## 1. Introduction

Mortality from advanced prostate cancer remains a significant clinical challenge worldwide. While several therapeutics including powerful androgen receptor (AR) pathway inhibitors (ARPi = AR-pathway inhibitor(s)), taxanes, and other approaches have improved prostate cancer management, end-stage castration-resistant disease remains incurable, highlighting the urgent need for the development of additional strategies to combat death from prostate cancer [1].

Poly (ADP-ribose) polymerase (PARP) inhibition is a precision treatment approach approved for use in patients harboring tumors with homologous recombination repair (HRR) deficiency [2,3,4]. While incompletely understood, PARP inhibitors (PARPi = PARP inhibitor(s)) are thought to work via inducing replication stress and accumulation of DNA double strand breaks [5,6,7,8]. Tumor cells deficient in HRR, such as those harboring BRCA1 and BRCA2 mutations, may inappropriately respond to PARPi-induced stress resulting in reduced viability. Based on results from the PROfound and TRITON2 clinical studies, PARPi monotherapy with olaparib or rucaparib, respectively, was approved for metastatic castration resistant prostate cancer (mCRPC) treatment in 2020 [9,10,11]. More recently, PARPi were approved in combination with ARPi [12,13]. Although PARPi and combinations have improved patient outcomes, treatment responses can be modest and eventual disease progression is common [14].

We hypothesize that improved understanding of responses to PARPi treatment may lead to co-targeting strategies that enhance and/or prolong therapeutic efficacy. While it is thought that PARPi-induced replication stress mediates much of the resulting cytotoxicity, the molecular response to the PARPi mechanism of action (MOA) is incompletely characterized. The DNA damage response (DDR) is complex, and further characterizing how DDR machinery orchestrates cellular response to PARPi-induced stress may shed light on vulnerabilities that can be co-targeted to exacerbate and enhance PARPi anti-tumor activity [15].

Despite initial favorable patient tumor responses to PARP inhibition, progression is both common and poorly understood [16]. It is thought that progression could be based on both (1) the selection of tumor cell sub-populations with intrinsic resistance and (2) the induction of adaptive programs which allow for survival under therapeutic pressure [17]. These avenues for progression may not be mutually exclusive, and adaptive mechanisms may range from alterations in cellular identity to changes in metabolism [18]. Understanding tumor cell adaptive responses may provide insight into the design of strategies to prolong treatment efficacy.

In the present study, we characterize response to PARP inhibition and investigate potential approaches which may improve treatment efficacy in CRPC. Treatment of PARPi-sensitive CRPC cells with a PARPi, olaparib, at different doses for both short and longer-term durations results in significant changes likely associated with both PARPi MOA and induction of downstream adaptive processes. Characterization of acute response to PARP inhibition reveals rapid and robust induction of cell cycle checkpoint signaling associated with activation of ATM. Co-targeting ATM with clinical stage ATM inhibitors dramatically improves response to PARP inhibition. At the later timepoint, we observe increased epithelial–mesenchymal transition (EMT)-associated gene expression which we hypothesize represents induction of a PARPi-adaptive mechanism. Targeting EMT via knockdown of EMT regulating transcription factor SLUG is shown to work in combination with olaparib in inhibiting cellular viability. Lastly, we also highlight PARPi-induced metabolic alterations which may also represent vulnerabilities. Altogether, this study advances our understanding of tumor cell responses to PARP inhibition and provides a foundation on which to further explore therapeutic strategies to improve patient outcomes.

## 2. Materials and Methods

### 2.1. Cell Culture, Reagents, and Treatments

C4-2B cells were kindly provided by Dr. Allen Gao (UC Davis) and authenticated via the ATCC’s STR profiling service. Abiraterone-resistant C4-2B-derived C4-2B-AbiR (AbiR) cells were acquired from the Gao Lab and previously described [19]. Briefly, C4-2B cells were chronically exposed to increasing doses of abiraterone over the course of ~12 months. All cell lines were routinely tested for mycoplasma contamination using the MycoStrip^®^—Mycoplasma Detection Kit (InvivoGen, San Diego, CA, USA, Cat#: rep-mys-20). Cell lines were kept frozen in liquid nitrogen for long-term storage and thawed out as needed. Experiments were conducted within 15 passages of resuscitation. All cells were maintained at 37 °C in a humidified incubator with 5% CO_2_ and cultured in RPMI-1640 medium (Corning, Manassas, VA, USA, Cat#: 10040CV) supplemented with 10% fetal bovine serum (Corning, collected in Mexico, processed in Woodland, CA, USA, Cat#: 35010CV) and 1% penicillin-streptomycin (Gibco, Grand Island, NY, USA, Cat#: 15140122). AbiR culture medium was additionally supplemented with 5 μM abiraterone acetate (Cat#: HY-75054), purchased from MedChemExpress (Monmouth Junction, NJ, USA). Olaparib (Cat#: S1060) was purchased from Selleck Chemical LLC. (Houston, TX, USA). Talazoparib (Cat#: HY-16106), Lartesertib (Cat#: HY-150617), AZD1390 (Cat#: HY-109566), Etomoxir (Cat#: HY-50202), and Fatostatin (Cat#: HY-14452) were purchased from MedChemExpress (Monmouth Junction, NJ, USA). All drugs were diluted in DMSO (Cat#: HY-Y0320) purchased from MedChemExpress (Monmouth Junction, NJ, USA). Knockdown of SNAI2 (SLUG) was performed using DsiRNA purchased from IDT (Newark, NJ, USA, Design ID: hs.Ri.SNAI2.13.2, Duplex sequences were 5′-ACUGAGUGACGCAAUCAAUGUUUAC-3′ and 5′-GUAAACAUUGAUUGCGUCACUCAGUGU-3′). Non-targeting negative control DsiRNA (IDT, Newark, NJ, USA, Cat#: 51-01-14-04) served as the control. Lipofectamine RNAiMAX (Invitrogen, Carlsbad, CA, USA, Cat#: 56532) was used for transfection of DsiRNAs. Images were taken with an Echo Revolve microscope (Discover Echo, San Diego, CA, USA) and analyzed with ImageJ (version 1.54r, National Institutes of Health, Bethesda, MD, USA) [20].

### 2.2. Cell Viability Assays

Cells were plated at 7500–20,000 cells/well in 24-well plates in complete media without selection agent 24 h prior to treatment. For cell growth assays involving RNAi and drug administration, siRNA transfection was performed 24hr after plating, followed by drug treatment the next day. Cell viability was assessed at indicated timepoints by either Cell Counting Kit-8 (CCK-8, Dojindo Molecular Technologies, Kumamoto, Japan, Cat#: CK04-20) or through the use of a Z1 particle counter (Beckman Coulter, Brea, CA, USA) to count cell number. CCK-8 procedure was previously described [21]. For synergy testing, cells were plated in a 96-well format and treated with a dose range matrix for 5-days. Viability was assayed using CCK-8. Synergy was analyzed with Combenefit (Combenefit, v.2.021, Cambridge, UK) [22]. The presented data are representative of three independent experiments.

### 2.3. RNA Sequencing Sample Preparation and RNA Isolation

C4-2B cells were plated and treated with DMSO or olaparib at 1 μM or 5 μM the following day and kept on treatment for either 1 or 5 days. Each condition (treatment + duration) was performed in triplicate for a total of 18 plated dishes. RNA isolation and quality checks were done as previously described [21]. RIN cutoff threshold was ≥7. All values attained for samples sequenced were ≥9 (Supplementary Table).

### 2.4. RNA-Seq, Data Analysis, and Bioinformatics

RNA sequencing and raw data processing were done as previously described [21]. Transcript-level abundance estimates were imported from Salmon’s native output files (quant.sf) using the tximport package in R, with type = “salmon” [23]. The tximport object was passed directly to DESeq2 via DESeqDataSetFromTximport to export two count matrices: an un-normalized matrix of gene-level estimated counts prior to normalization, and a normalized matrix [24]. RNA-seq data are available via the GEO database (Accession#: GSE322615) [25].

Downstream analysis was performed in R (v4.4.1) starting from the gene-level raw count matrix. The RNA sequencing data were quality checked and post-processed using DESeq2 v1.40.2 [24]. Euclidean distances were calculated on variance-stabilized values using a parametric fit and clustered using hclust from the stats v3.6.2 package. Differential expression analysis was carried out using a local regression fit. Cutoffs applied for differentially expressed genes displayed in UpSet and volcano plots were adjusted *p*-value < 0.05 and |log2(Fold Change)| > 0.5. GSEA was performed on all significant (adjusted *p*-value < 0.05) differentially expressed genes using clusterProfiler v4.8.3 [26,27,28]. All code used in this project is freely available on Github (https://github.com/marionhardy/AK_C42B_LLG001-018 (accessed on 15 April 2026)).

### 2.5. Western Blotting

Whole cell lysates were prepared and protein concentrations checked as previously described [21]. Proteins were resolved via SDS-PAGE. Primary antibodies purchased from Cell Signaling (Danvers, MA, USA) used for protein detection are as follows: PAR (Cat#: 83732), γ-H2AX (Cat#: 9718), p21 (Cat#: 2947), cleaved-PARP (Cat#: 9541), Vimentin (Cat#: 5741), SLUG (Cat#: 9585), β-Tubulin (Cat#: 2128), ATM (Cat#: 2873), phospho-ATM Ser1981 (Cat#: 13050), p53 (Cat#: 9282), and phospho-p53 Ser15 (Cat#: 9284). Loading was checked with Ponceau S staining (Tocris Bioscience, Bristol, United Kingdom, Cat#: 5225) and Tubulin. All blot panels with a corresponding tubulin derive from a single set of protein samples. Proteins were detected via chemiluminescence (MilliporeSigma, Burlington, MA, USA, Cat#: WBLUF0500) using a ChemiDoc MP Imaging System (Bio-Rad, Hercules, CA, USA). The displayed data are the chemiluminescent blots. Also provided in the Supplementary Materials are uncropped composite blots which are compositions of the chemiluminescent and colorimetric blot data. All blots are representative of at least three independent experiments and are intended to be semi-quantitative evidence.

### 2.6. Flow Cytometry

Cell-cycle distribution was assessed via flow cytometry with a BD LSRII flow cytometer using propidium iodide (PI) from the Propidium Iodide Flow Assay Kit (EnQuire BioReagents, Littleton, CO, USA; Cat#: Qkit33-200T). Cells were plated and treated 24 h later. After 5 days, cells were fixed in 66% ethanol solution and stored (up to 4 weeks in accordance with manufacturer’s protocol) at 4 °C until PI staining was performed according to manufacturer’s protocol. Stained cells were subjected to flow cytometry detection, and data were analyzed via FloJo software (10.10.1) utilizing the cell cycle module (Univariate modeling: Watson (Pragmatic) parameters). All conditions were performed in triplicate. The presented data are representative of three independent experiments.

### 2.7. 3D-Bioprinted Tumoroid Drug Testing

A patient-derived prostate tumor specimen was functionally evaluated using a 3D-bioprinted tumoroid platform designed for high-throughput drug response profiling. The specimen was collected under UC Davis IRB-approved protocol (ID: 222924-31, UC Davis GenitoUrinary (GU) Biospecimen Collections (Tissue, Blood and Urine)) and the patient provided informed consent. Fresh tumor tissue was mechanically and enzymatically dissociated into a single-cell suspension using the gentleMACS Dissociator (Miltenyi Biotec, Bergisch Gladbach, Germany), preserving heterogeneous cellular components of the tumor microenvironment. The resulting cell suspension was embedded in a Type I/III collagen-based extracellular matrix and bioprinted into a 96-well plate as ~1.5 µL droplets to generate uniform 3D tumoroid constructs using a Bio One bioprinter (Cellink, Gothenburg, Sweden). Constructs were overlaid with complete media as described, supplemented with 1 nM DHT and exposed to either olaparib (10 μM), AZD1390 (10 nM), or a combination [29]. Treatments were performed in triplicate. To incorporate immune context beyond tumor-intrinsic immune cells, tumoroids were co-cultured with autologous peripheral blood mononuclear cells (PBMCs) isolated from matched patient blood samples at a 30:1 ratio. Co-cultures were maintained for 6 days under controlled conditions to allow tumor–immune interactions and drug responses to manifest. At endpoint, tumoroid viability was assessed using a 3D-compatible live/dead fluorescence assay (Cyto3D), followed by high-content imaging using an Agilent BioTek Cytation 5 multimode reader (Agilent Technologies, Santa Clara, CA, USA). Image-based quantification was performed to capture both viable tumor burden and cell death. A composite response score was calculated to integrate cytostatic and cytotoxic effects, using a weighted model of growth inhibition and cell death. Scores were normalized to control conditions, with more negative values indicating greater anti-tumor efficacy. Comparative analyses were performed across treatment conditions to assess differential drug sensitivity.

### 2.8. Statistics and Schematics

Statistics and graphing were done with GraphPad Prism (v.10; GraphPad Software, Boston, MA, USA) unless otherwise noted. Significance was calculated using Welch’s unpaired two-tailed *t*-test or ordinary one-way ANOVA followed by Dunnett’s or Sidak’s multiple comparison tests, as appropriate and indicated in the figure legends. Significance is reported as ns = not significant, * = *p*-value ≤ 0.05, ** = *p*-value ≤ 0.01, *** = *p*-value ≤ 0.001, unless otherwise noted in the figure legend.

## 3. Results

### 3.1. A Dichotomy Is Observed Regarding CRPC Cell Response to PARP Inhibition

**Figure 1:**
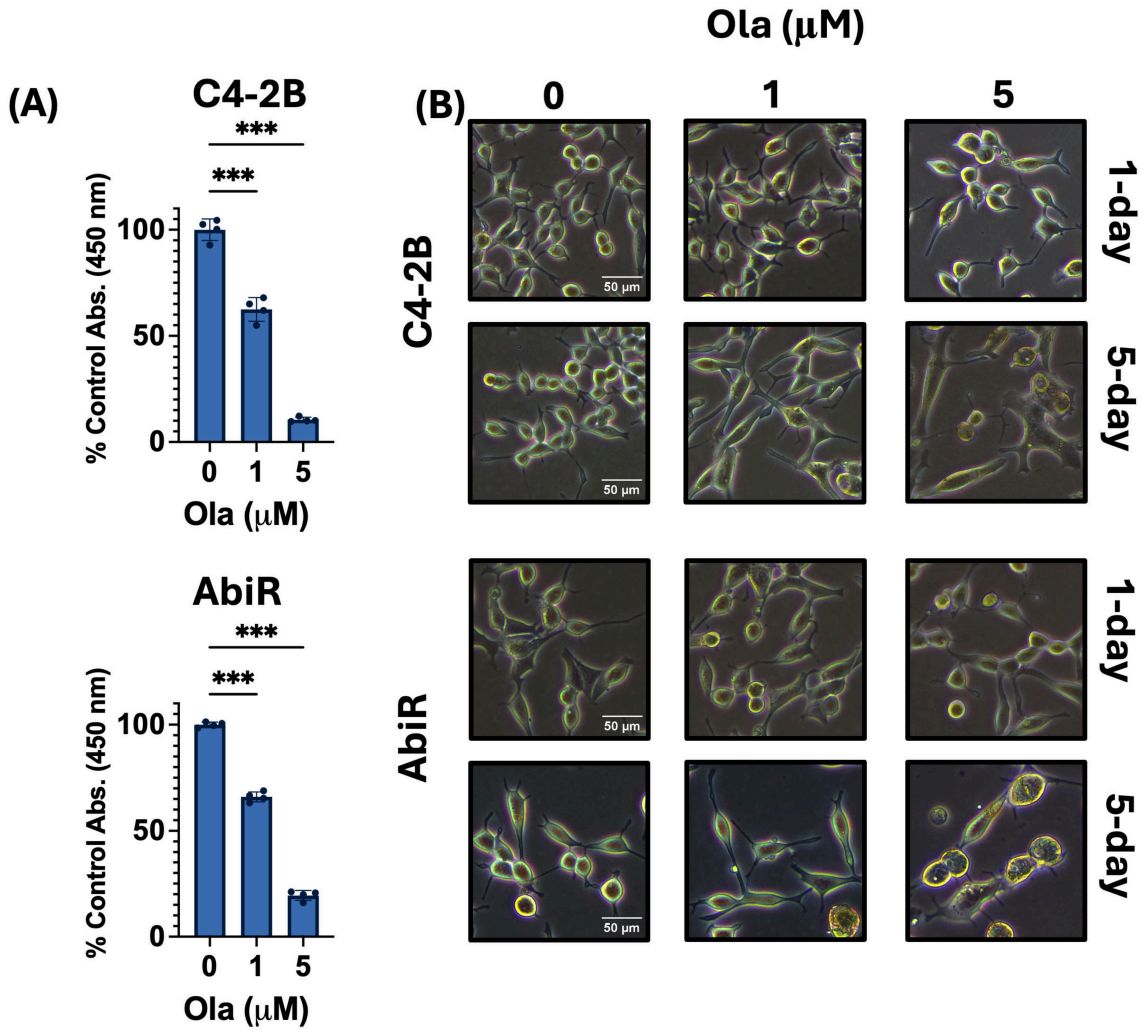
Assessment of response to olaparib in CRPC cells. (**A**) Cell viability assays show sensitivity of C4-2B and AbiR cells to olaparib treatment at 5 days via CCK-8. Data are presented as a % of control viability +/− standard deviation. Data were analyzed using ordinary one-way ANOVA with Dunnett’s multiple comparison test (*n* = 4 replicates/condition). (**B**) Phase contrast microscopy reveals morphology of treated C4-2B and AbiR cells with indicated doses of olaparib at indicated timepoints. Scale bars represent 50 µm and apply to all panels. All images were acquired using identical magnification. All data are representative of three independent experiments. *** = *p*-value ≤ 0.001. Ola = olaparib.

We subjected the mCRPC cell line, C4-2B, and its abiraterone-resistant derivative, C4-2B-AbiR (AbiR), to cell viability assays testing treatment with olaparib for five days. Dosing was guided by prescribing information and previous reports of olaparib pharmacokinetics [30]. C4-2B cells have been shown to respond to clinically relevant PARPi dosing and are derived from PARPi-sensitive LNCaP cells which were shown to possess mutations in HRR-associated genes [31,32,33]. Both C4-2B and AbiR demonstrated sensitivity to clinically relevant doses of olaparib (Figure 1A). At the conclusion of the experiment, we observed notable morphological changes in the surviving subset of cells at both concentrations, with more pronounced effects at the 5 µM dose. To further assess the impact of treatment on morphology, both cell lines were treated with 1 µM or 5 µM olaparib for either 1 or 5 days and imaged (Figure 1B). After one day of treatment, the cells largely retained parental morphology. In contrast, longer exposure and higher doses promoted a striking shift in appearance in the remaining cells, which exhibited a range of characteristics, including those associated with senescence as well as enlargement and elongation. Similar findings were observed in C4-2B cells in response to talazoparib, another clinically relevant PARPi (Supplementary Figure S1).

Our observations highlight a dichotomy regarding thinking about cell response to treatment which may support two ways to design co-targeting strategies with a given drug. The first would be to target and seek to exacerbate the drugs MOA. Though not readily observable, PARPi are thought to elicit their mechanistic effects on cells on the order of minutes and hours [5,8]. Understanding early molecular response to treatment may provide opportunities to enhance PARPi anti-tumor effects by working to exploit the specific actions of the drug. The second strategy would seek to enhance treatment durability by targeting adaptation. Morphological alterations observed after prolonged exposure may in part be associated with induction of adaptive mechanisms. Targeting these may be a means to extend treatment efficacy. Investigating response with respect to time may provide an avenue to investigate crucial alterations which mediate initial effects and those that promote cell survival on protracted treatment. Combining these insights could ultimately provide co-targeting strategies that can synergize with treatment or be utilized to hinder the viability of drug-tolerant populations.

### 3.2. RNA Sequencing Reveals Time- and Intensity-Dependent Differences in PARPi Treatment Responses

**Figure 2:**
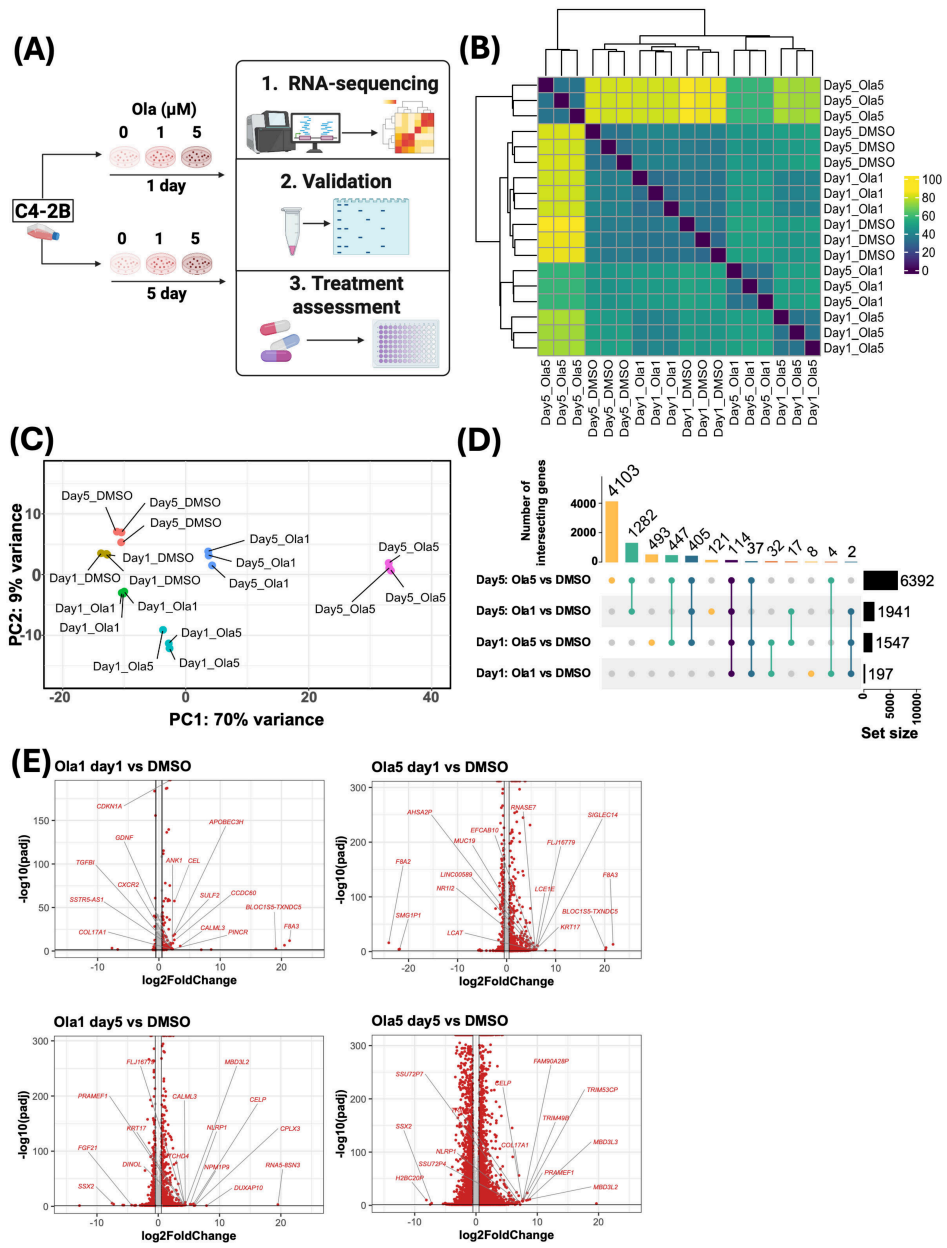
RNA sequencing reveals transcriptomic changes associated with response to olaparib in C4-2B cells. (**A**) Schematic of study design. Triplicate C4-2B samples were subjected to indicated treatments for either 1 or 5 days. RNA sequencing and analysis were performed followed by validation and testing of potential therapeutic strategies. Created in BioRender. Hardy, M. (2026) https://BioRender.com/2c4fswu (accessed 15 April 2026). (**B**) Euclidean distance plotting reveals transcriptomic changes in response to indicated treatment doses and durations. (**C**) Principal component analysis (PCA) reveals variance between samples receiving different treatment intensity and duration. (**D**) UpSet plot reveals differentially expressed genes (DEG) by treatment (set size) and both uniquely specific and shared DEGs across treatments (intersecting genes—indicated by connected colored dots across groups). (**E**) Volcano plots display DEGs plotted by expression (log2foldchange) versus statistical significance (–log10(padj)). Ola = olaparib.

We sought to improve understanding of cellular response to PARP inhibition to guide rational design of combination treatments. To investigate responses to differential treatment exposure, triplicate C4-2B samples were subjected to either vehicle treatment or olaparib 1 µM or 5 µM for a duration of either 1 or 5 days and submitted for RNA sequencing (Figure 2A). Graphing Euclidean distances suggests that the intensity of treatment drives increased transcriptional change (Figure 2B). Principle component analysis supports this observation but provides additional depth of information (Figure 2C). While intensity appears to largely be captured in principle component 2 (PC2, y-axis), the duration of treatment drives more dramatic change captured largely in PC1 (x-axis). Treatments at 5 days exhibit more change along PC1 than PC2, suggesting changes associated with time are more significant contributors to surviving cell phenotype. An UpSet plot, which captures both treatment specific and intersection specific changes, further supports that both intensity and time on treatment drive transcriptional change, with 5-day treated samples exhibiting much greater numbers of significantly differentially expressed genes (DEG) versus respective 1-day treatments (Figure 2D). This observation is further supported with volcano plots displaying DEGs (Figure 2E).

**Figure 3:**
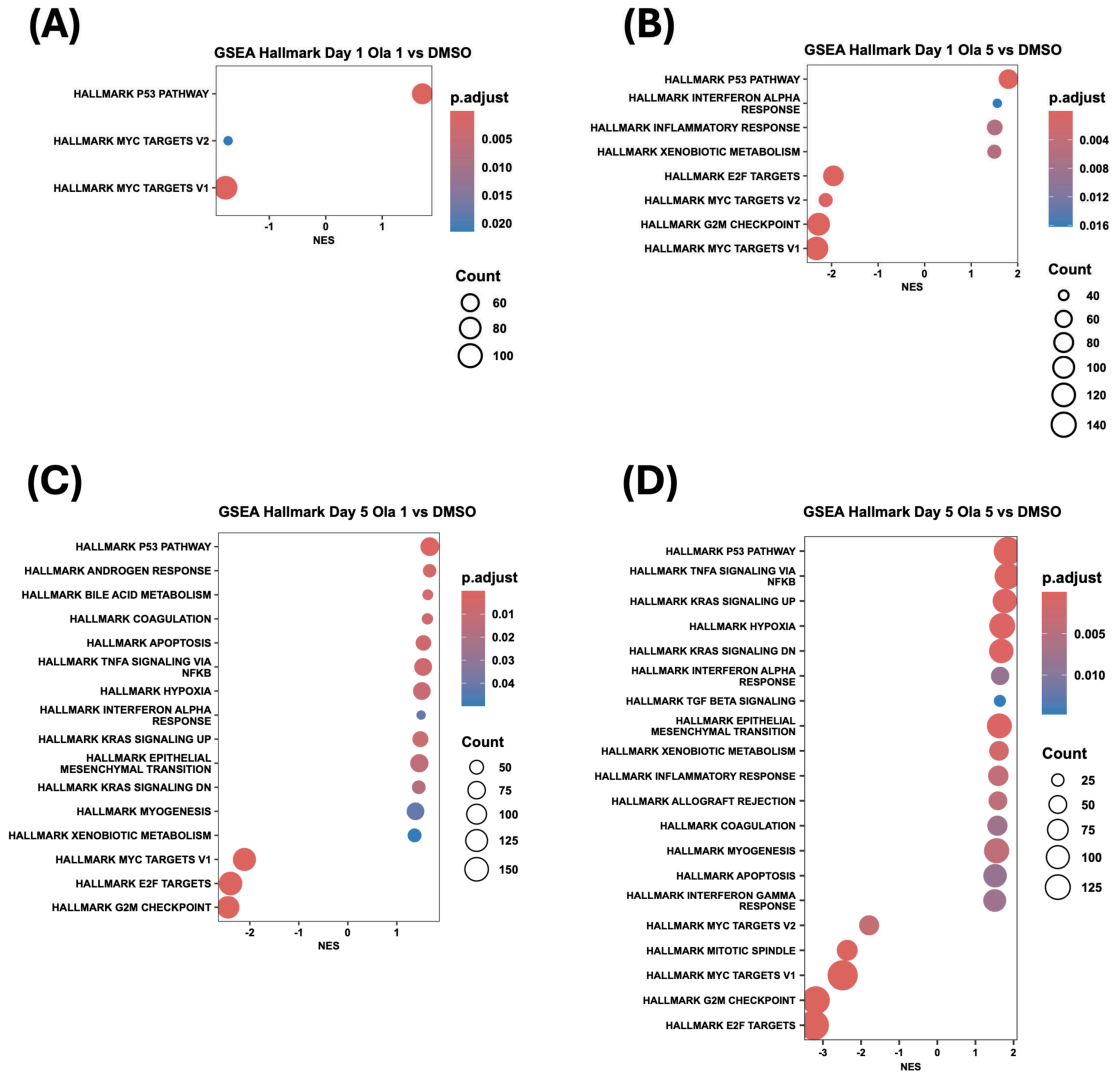
Gene set enrichment analysis of Hallmark gene sets in C4-2B cells treated with 1 μM or 5 µM olaparib for 1 or 5 days. GSEA dot plots displaying significantly altered Hallmark pathways comparing olaparib (Ola) treated cells to respective vehicle (DMSO) controls. Results are displayed for (**A**) 1 day, Ola 1 µM, (**B**) 1 day, Ola 5 µM, (**C**) 5 days, Ola 1 µM, (**D**) 5 days, Ola 5 µM. Up to the top 20 most significant pathways by padj are displayed for each experimental condition.

We next subjected DEGs to gene set enrichment analysis (GSEA) to investigate pathway-level changes in response to differences in both treatment intensity and duration. Hallmark pathway analysis reveals enrichment of the p53 signaling pathway as the most upregulated significant gene set for both treatments at day 1 (Figure 3A,B). We additionally find significant downregulation of cell-cycle-related gene sets in response to both doses. This implies that initial response to PARP inhibition involves activation of cell cycle checkpoint signaling in line with accepted PARPi MOA. Interrogation of the GO biological process (GO:BP) and Reactome pathways at the 1-day timepoint largely points to downregulation of translational processes as well as decreased gene expression associated with DNA organization, epigenetics, and RNA processing, while further supporting decreased cell-cycle-related signaling (Supplementary Figures S2A,B and S3A). Notably, 5 μM olaparib 1-day treatment also significantly increases gene sets associated with immune signaling and xenobiotic metabolism (Figure 3B).

At day 5, we show evidence for sustained p53 signaling, as well as more pronounced downregulation of cell-cycle-related gene sets at both doses relative to Day 1 (Figure 3). Cell-cycle-related results are further supported by interrogation of GO:BP and Reactome gene sets (Supplementary Figures S2 and S3). We also observe significant upregulation of apoptosis-associated signaling at day 5 (Figure 3C,D). Together, these data imply that prolonged PARPi exposure leads to sustained activation of checkpoint signaling which may lead to both cell death and a surviving fraction with reduced proliferative capacity.

We hypothesize that enrichment of additional pathways may be involved in adaptive processes. Notably, the hallmark epithelial–mesenchymal transition (EMT) gene set is significantly elevated at both doses at day 5 relative to day 1 (Figure 3). Enrichment of gene expression associated with extracellular matrix organization further supports acquisition of an EMT phenotype (Supplementary Figure S3B,C). EMT is known to be associated with the development of treatment resistance [34]. This finding supports observed morphology of persisting cells and suggests EMT may be induced to promote survival. Altogether, RNA sequencing reveals critical insight regarding both short- and longer-term response to PARP inhibition. We sought to test whether this information could inform the design of combination treatment strategies.

### 3.3. Targeting ATM-Dependent DNA Damage Response Significantly Enhances Efficacy of PARPi Treatment

**Figure 4:**
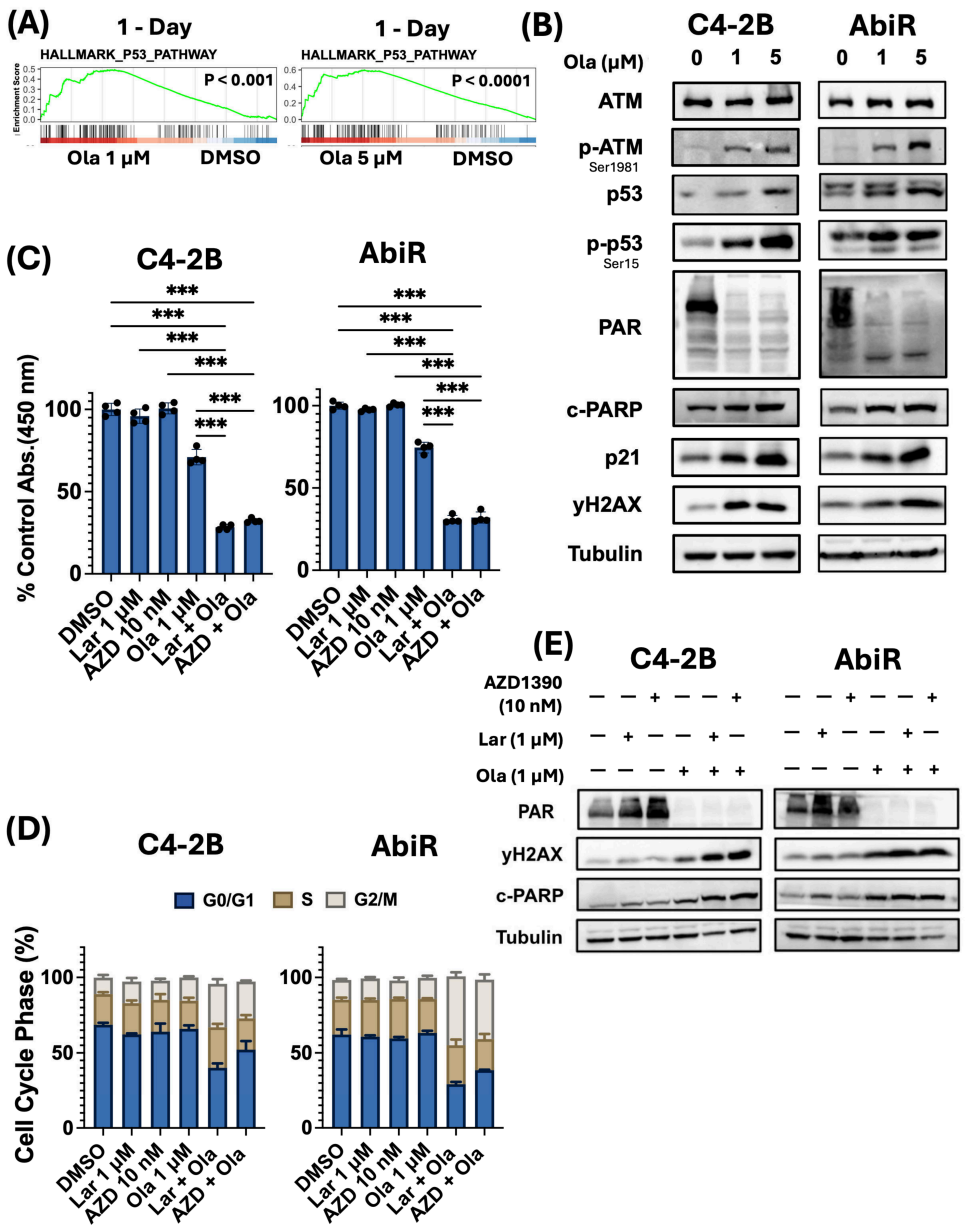
ATM inhibition significantly enhances olaparib response in C4-2B and AbiR cells. (**A**) GSEA plots displaying hallmark p53 pathway gene set in response to olaparib 1 µM or olaparib 5 µM after 1 day of treatment in C4-2B cells. (**B**) Western blots reveal expression of indicated protein markers in response to olaparib at the indicated dosages after 1 day of treatment. Tubulin served as a loading control. (**C**) Cell viability assays show response of C4-2B and AbiR cells after 5 days of indicated treatments via CCK-8. Data are presented as a % of control viability +/− standard deviation. Data were analyzed using ordinary one-way ANOVA with Sidak’s multiple comparison test (*n* = 4 replicates/condition). (**D**) Bar graphs demonstrate cell cycle distribution after 5 days of indicated treatments. (**E**) Western blots reveal expression of indicated protein markers in response to presence (+) or absence (−) of indicated treatments after 5 days. Tubulin served as a loading control. *** = *p*-value ≤ 0.001. Ola = olaparib. Lar = lartesertib. AZD = AZD1390. All data are representative of three independent experiments.

We reasoned that transcriptomic changes significantly altered at the 1-day timepoint are largely indicative of acute effects associated with PARPi MOA. As noted above, these early changes appear largely characterized by increased p53 signaling and decreased cell-cycle-associated gene expression, suggesting rapid induction of DNA damage and activation of the DDR (Figure 4A). In support of RNA-seq data, western blots show increased expression of γH2AX (DNA damage marker) and phosphorylated p53 (Ser15) in both C4-2B and AbiR cells undergoing 1 μM or 5 μM olaparib treatment for 24 h (Figure 4B). In line with increased p53 signaling, we also see higher expression of both p21 and cleaved-PARP (c-PARP), indicative of cell cycle arrest and cell death respectively. These findings support current understanding of PARPi MOA and our previous report [32].

The DDR is largely governed by members of the PI3K-related kinase family, including ATM [35]. DNA damage promotes activation of ATM which is autophosphorylated at Ser1981 [36]. Indeed, 24 h olaparib treatment greatly increases expression of phosphorylated ATM in both C4-2B and AbiR cells, suggesting its involvement in response to PARPi-induced stress (Figure 4B). Given ATM’s described function in coordinating the DDR, we hypothesized that ATM inhibition would impair tumor cell response to PARPi treatment and enhance efficacy. Using two clinical stage ATM inhibitors (ATMi = ATM inhibitor(s)), Lartesertib (M4076) and AZD1390, we show that both combined with olaparib significantly reduces cell viability in both C4-2B and AbiR cells (Figure 4C) [37]. To test whether a PARPi and ATMi combination may achieve synergy, we utilized Bliss synergy modeling to assess combination of lartesertib with olaparib in C4-2B cells and found direct evidence for synergy across a range of doses between these agents (Supplementary Figure S4A). ATMi combination with talazoparib similarly significantly reduces C4-2B cell viability versus monotherapy (Supplementary Figure S4B,C).

Next, we sought to further investigate the efficacy of combining PARPi and ATMi in reducing tumor cell viability. We performed flow cytometry to investigate changes in cell cycle distribution in response to monotherapy or combination treatment (Figure 4D and Figure S4D). In agreement with a previous report, combination treatment led to marked increases in cells in the G2/M phase in both cell lines [38]. Western blots suggest that olaparib-induced yH2AX and c-PARP expression is enhanced by combination treatment with either M4076 or AZD1390, which support increased DNA damage and cell death, respectively (Figure 4E). Altogether, the data support that PARP inhibition induces DNA damage and an ATM-dependent DDR which can be targeted to increase DNA damage and the efficacy of treatment. ATM inhibition may be a promising strategy for increasing PARPi utility through targeting the PARPi MOA.

### 3.4. Prolonged PARPi Treatment Promotes an EMT Phenotype Which May Promote Adaptation

**Figure 5:**
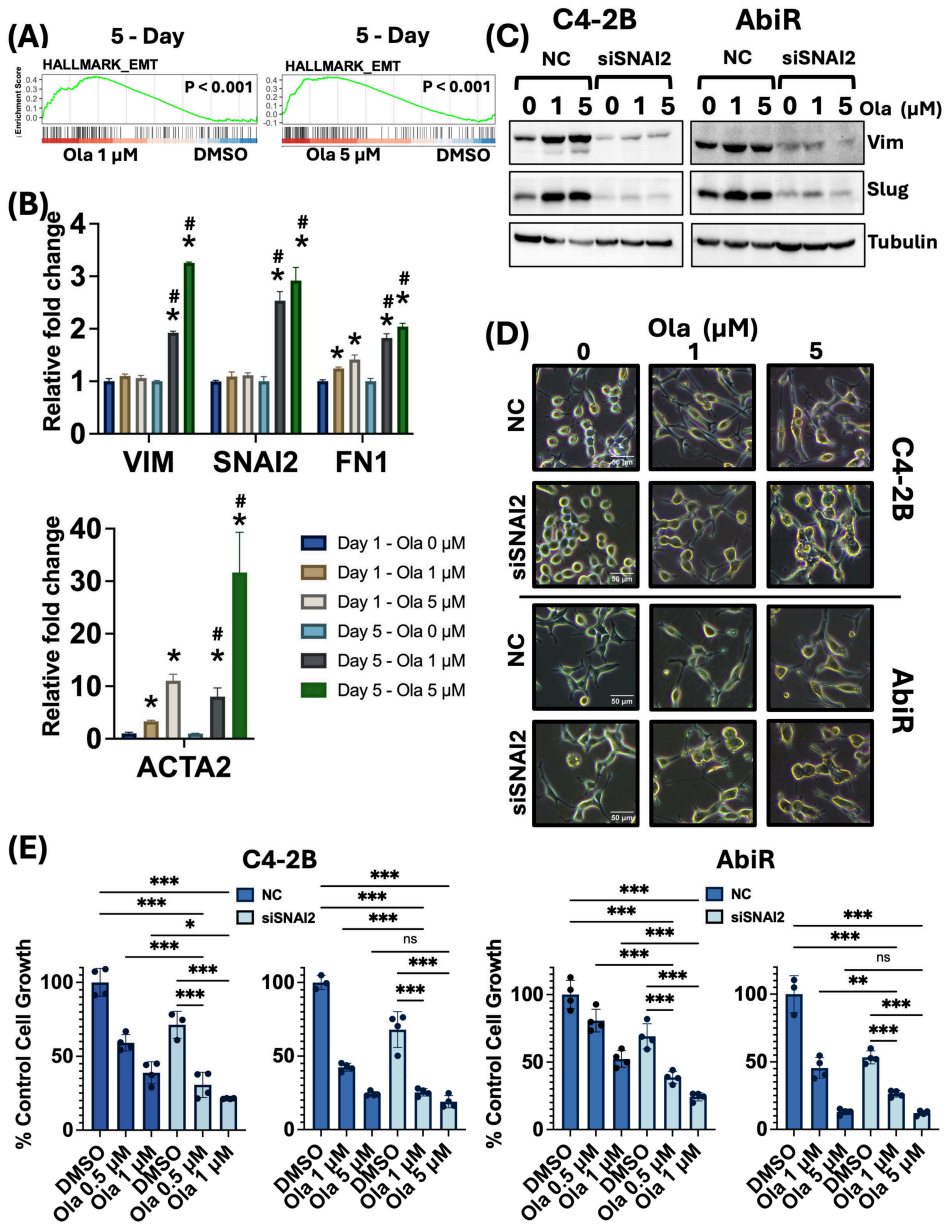
Prolonged PARP inhibition promotes the EMT phenotype in C4-2B and AbiR cells. (**A**) GSEA plots displaying the hallmark EMT pathway gene set in response to either olaparib 1 µM or olaparib 5 µM after 5 days of treatment in C4-2B cells. (**B**) Analysis of RNA-seq read counts (averages of groups normalized to control) for indicated EMT-related genes in C4-2B cells at indicated doses of olaparib for either 1-day or 5-day treatment. * indicates significance (*p* ≤ 0.05) as compared to respective control. # indicates significance (*p* ≤ 0.05) comparing 5-day to respective 1-day treatments. Data were analyzed with two-tailed, unpaired *t*-test with Welch’s correction (*n* = 3 replicates/condition). (**C**) Western blots reveal expression of indicated EMT-related genes in response to olaparib treatment in C4-2B and AbiR cells after 5 days of treatment with or without SNAI2 knockdown. Tubulin served as a loading control. (**D**) Phase contrast microscopy reveals morphology of treated C4-2B and AbiR cells with indicated doses of olaparib after 5 days of treatment with or without SNAI2 knockdown. Scale bars represent 50 µm and apply to all panels. All images were acquired using identical magnification. (**E**) Cell viability assays show sensitivity of C4-2B and AbiR cells to 5-day olaparib treatment at indicated dosages with or without SNAI2 knockdown via coulter counter. Data are presented as a % of control viability +/− standard deviation. Data were analyzed using ordinary one-way ANOVA with Sidak’s multiple comparison test (*n* = 4 replicates/condition). * = *p*-value ≤ 0.05, ** = *p*-value ≤ 0.01, *** = *p*-value ≤ 0.001, ns = not significant. Ola = olaparib. NC = non-targeting control siRNA. siSNAI2 = SNAI2 targeting siRNA. EMT = epithelial–mesenchymal transition. All data are representative of three independent experiments.

Our findings support a model whereby PARP inhibition induces cell cycle checkpoint activation in response to DNA damage. However, although we observe cell death, we also note survivors with greatly altered morphology at the later 5-day timepoint. This suggests that a subset of cells persist and may begin to adapt over time. Notable morphologic observations included cell enlargement and elongation, which suggest phenotypic shifts. GSEA reveals significant enrichment of the hallmark EMT gene set at both treatment doses at 5 days (Figure 3C,D and Figure 5A). These data are in line with observed treated cell morphology and suggest EMT induction may be involved in adaptation to treatment. Genes driving enrichment include well characterized EMT markers such as VIM, FN1, and ACTA2, and the EMT regulating transcription factor SNAI2 (SLUG) [39]. All four of these genes are significantly elevated at both Ola 1 µM and 5 µM doses at 5 days versus 1 day (Figure 5B). EMT is thought to be associated with cellular plasticity and resistance to treatment [40]. We hypothesized that increased expression of the EMT promoting transcription factor SLUG induces EMT in response to PARP inhibition leading to treatment insensitivity and survival in persisting cells. Western blots support increased expression of SLUG and VIM in response to 5-day olaparib treatment in both C4-2B and AbiR cells (Figure 5C). SLUG knockdown results in decreased expression of SLUG and VIM and decreased appearance of mesenchymal features in PARPi-treated cells (Figure 5C,D). These data support the hypothesis that upregulated SLUG drives EMT in response to prolonged olaparib exposure. To initially test whether SLUG-driven EMT affects drug tolerance, we knocked down SLUG in both C4-2B and AbiR cells and followed with olaparib treatment for 5 days. Decreasing EMT through SLUG knockdown resulted in decreased viability of tumor cells in combination with olaparib versus monotherapies (Figure 5E). This suggests that PARPi-induced EMT may in turn provide tolerance for cells to persist on treatment. Targeting EMT may be a strategy for enhancing and/or prolonging treatment response. How inhibition of EMT affects long-term progression remains to be tested.

### 3.5. Olaparib-Induced Metabolic Alterations May Represent Targetable Vulnerabilities

**Figure 6:**
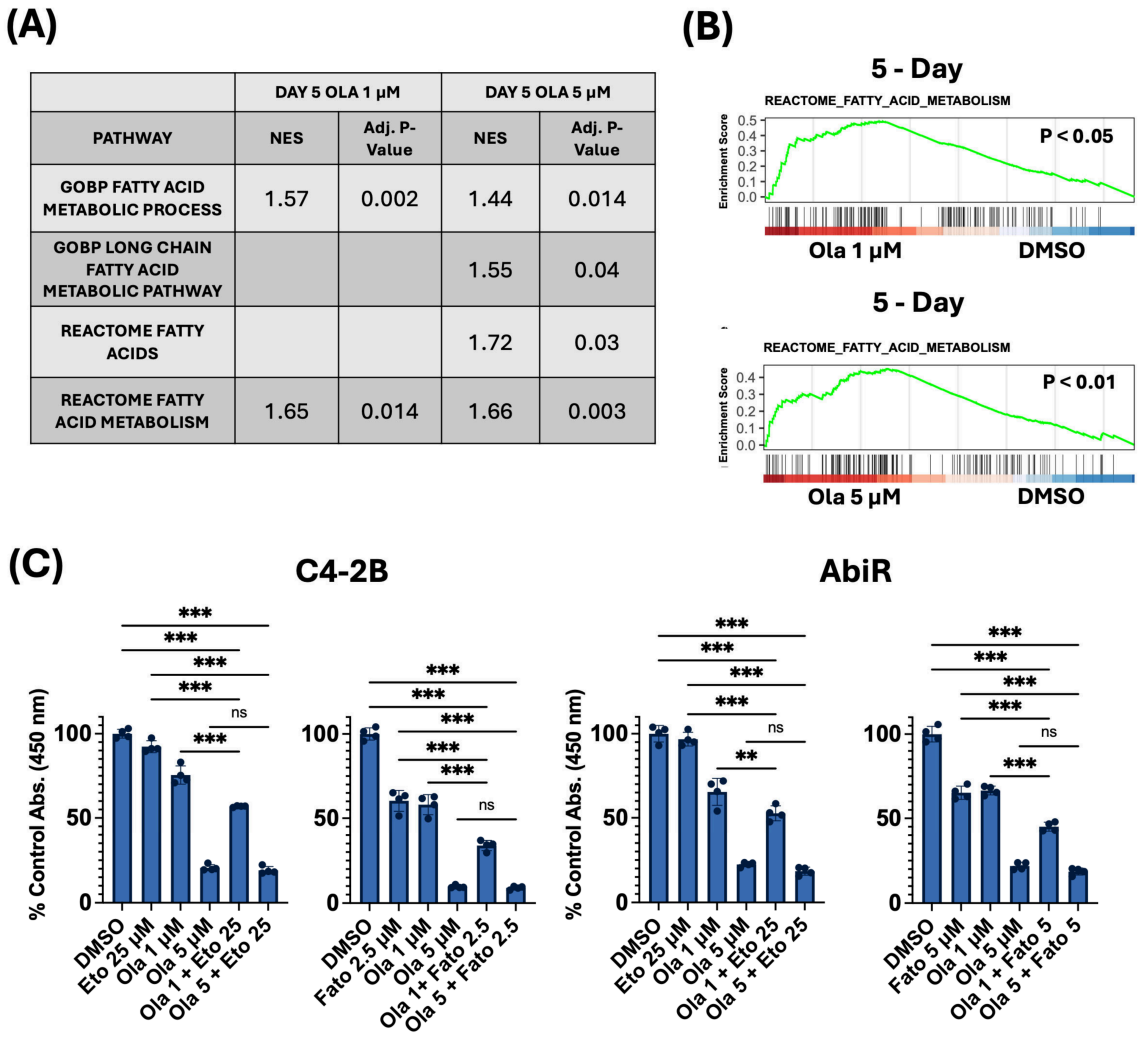
PARP inhibition may induce adaptive alterations in fatty acid metabolism. (**A**) Significantly enriched FAM-associated pathways from reactome and GO:BP GSEA gene sets displayed with normalized enrichment scores (NES) and *p*-adj in C4-2B cells. (**B**) GSEA plots displaying reactome FAM pathway gene set in response to either olaparib 1 µM or olaparib 5 µM after 5 days of treatment in C4-2B cells. (**C**) Cell viability assays show sensitivity of C4-2B and AbiR cells to fatty acid oxidation inhibitor etomoxir (Eto) or fatty acid synthesis inhibitor fatostatin (Fato) with or without indicated olaparib dosing after 5-day treatment. Data are presented as a % of control viability +/− standard deviation. Data were analyzed using ordinary one-way ANOVA with Sidak’s multiple comparison test (*n* = 4 replicates/condition). ** = *p*-value ≤ 0.01, *** = *p*-value ≤ 0.001, ns = not significant. Ola = olaparib. Eto = etomoxir, Fato = fatostatin. All data are representative of three independent experiments.

Further interrogation of RNA-seq data suggests significant metabolic alterations occur in response to PARP inhibition. Notably, we found evidence for enrichment of pathways related to fatty acid metabolism (FAM) (Figure 6A,B). The DDR is thought to be energetically costly, and it is hypothesized that catabolic processes like fatty acid oxidation (FAO) may be needed to provide energy for responding to DNA damage [41]. These data suggest that persisting tumor cells may exhibit a shifted metabolism which promotes response to PARPi-induced damage. We hypothesize this shift may represent a targetable vulnerability. FAM includes both FAO and fatty acid synthesis (FAS), both of which are thought to be able to coexist and support one another within the same cellular context [42]. To investigate potential reliance on altered FAM, we investigated olaparib combinations with either an inhibitor of FAO (etomoxir) or FAS (fatostatin) (Figure 6C). Combinations of either drug with 1 μM olaparib exhibited significant cell growth inhibition compared to respective monotherapies, while the 5 μM dose of olaparib appeared too effective on its own to reveal a combination effect. These data imply that targeting altered metabolic phenotypes induced by PARP inhibition may enhance efficacy of treatment. How combinations may alter long-term progression on a PARPi remains to be tested.

## 4. Discussion

PARP inhibition has improved the management of advanced malignancies including those of the prostate, ovary, breast, and pancreas [43]. Still, disease eventually progresses on PARPi, necessitating the development of strategies to increase and prolong treatment efficacy [44]. There is much we do not understand about cellular response to treatment and eventual adaptation. The current study sought to use models of PARPi-sensitive prostate cancer to explore how characterization of both short- and longer-term treatment may inform the design of improved PARPi regimens.

Our findings highlight a dichotomy in thinking about how to design combination therapeutic strategies. Assessment of response to treatment at early timepoints is expected to inform largely on direct cellular response to the actions of the therapy. These effects may not be phenotypically observable but insight into their molecular underpinnings may reveal exploitable targets. While cell morphology is largely unchanged 1 day post olaparib treatment, RNA sequencing reveals transcriptomic changes aligned with the expected MOA of PARP inhibition; notably, evidence for activation of the DDR, cell cycle checkpoint signaling, and p53. The DDR and downstream signaling is governed largely by members of the PI3K-like kinase (PIKK) family of kinases, which include ATR, DNA-PK, and ATM [35]. Western blots for ATM Ser1981 phosphorylation suggest early activation of ATM in response to PARPi, and ATM has been shown to phosphorylate p53 at ser15 [45,46]. We hypothesized that targeting ATM would impair cellular response to PARP inhibition and exacerbate treatment-induced cytotoxicity. Indeed, use of two clinical stage ATMi dramatically enhanced the efficacy of both olaparib and talazoparib. To our knowledge, C4-2B cells possess at least one intact allele of ATM, supporting our observation of ATMi efficacy [33]. These findings highlight the potential of combining PARPi with inhibitors of DDR regulators. Previous studies in other cancer contexts have shown that ATM inhibition may potentiate PARPi effectiveness [37]. AZD0156 was shown to increase PARPi sensitivity in diverse cancer models (head and neck, non-small-cell lung, breast, gastric) while M4076 demonstrated strong activity in combination with either rucaparib or niraparib in a breast cancer model [38,47]. Additionally, our data support previous work which demonstrated synergy between an ATMi and a PARPi [48]. The specific mechanism underlying combination utility remains to be fully elucidated. We present evidence that combination treatment leads to increased DNA damage (higher yH2AX levels), G2/M cell cycle checkpoint activation, and further increases in cell death. These data support a model whereby ATM responds to PARPi-induced DNA damage which is worsened by co-inhibition of ATM. Mak et al. proposed a model whereby ATMi-induced DNA damage is exacerbated by PARP inhibition leading to G2 cell cycle checkpoint activation [48]. Aberrant entry into mitosis then results in mitotic and post-mitotic cell death. In support of this model, our team has observed that reduced viability of a patient-derived prostate tumoroid model using AZD1390 may be further decreased in combination with olaparib (Supplementary Figure S5). However, this finding derives from a single patient sample and should be interpreted as exploratory data supporting the hypothesis that PARPi and ATMi may be combined to treat advanced prostate cancer. Both models support efficacy of combining PARPi and ATMi, but the specific mechanistic basis for increased DNA damage requires further elucidation. Evidence suggests that PARPi efficacy may in fact rely upon defects in replication single strand DNA gap suppression rather than HRR, and loss of ATM is thought to result in gap accumulation [49,50,51,52]. Thus, combination efficacy may be due to PARPi-mediated exploitation of ATMi-induced defective gap suppression, but this hypothesis remains to be tested in future work. Altogether, these data support the overarching hypothesis that improved understanding of treatment response can lead to the design of increasingly efficacious therapeutic strategies such as combination with ATM inhibition. Future work should be directed at understanding how responses differ based on different genetic backgrounds, including various PARPi sensitizing lesions, and mutation of p53, which occurs frequently in advanced tumors [53,54].

It is important to note that higher dosing (5 μM versus 1 μM) led to more dramatic changes. While expected, this insight is important when considering the utility of combinations. Due to dosing regimens and spatial organization within a tumor, it is thought that tumor cells are variably exposed to therapy [55]. Thus, not every cell would be expected to experience higher, more efficacious concentrations of treatment. Efforts to understand and exploit tumor cell therapeutic response are needed to identify synergistic strategies which may significantly increase utility. Work presented shows that combination of an ATMi with a PARPi may synergistically decrease tumor cell viability. These data suggest these combinations may augment exposure to effective treatment concentrations resulting in better disease control. Still, PARPi treatment with either higher doses or combination with an ATM inhibitor resulted in the emergence of a surviving persister cell population upon prolonged exposure, necessitating the need to investigate mechanisms which enable adaptation.

While earlier transcriptomic changes are more likely to directly point toward cellular response to treatment MOA, changes at later timepoints may more readily inform on induction of adaptive programs. Surviving cells at day 5 post treatment exhibit significantly altered morphology implying phenotypic shifts in response to PARP inhibition. Notably, we observed that EMT signaling becomes significantly enriched at day 5 in response to either 1 μM or 5 μM olaparib. These data support observed cellular morphological changes. EMT is often associated with enabling metastatic spread of tumor cells [56]. However, EMT has also been linked to treatment insensitivity [57]. Our data show that inhibition of SLUG-dependent EMT may potentiate effects of olaparib which supports a role for EMT induction in promoting survival, adaptation, and resistance to treatment. Franca et al. demonstrated that overexpression of SLUG may directly confer resistance to olaparib in an ovarian cancer cell line model [55]. Additionally, EMT is thought to underly cellular plasticity and acquisition of stemness which can promote progression [58,59,60]. Emerging evidence also links EMT to development of drug-tolerant persister states which may drive treatment failure [61]. Interestingly, Franca et al. note that EMT may both directly promote treatment insensitivity and adaptation over time. Future work is needed to investigate how EMT promotes long-term adaptation to PARP inhibition. It is possible that SLUG promotes PARPi insensitivity in an EMT-independent manner. This will also be addressed in future work.

Further interrogation of our sequencing data revealed additional potential adaptive mechanisms, including changes in cell metabolism. Notably, we found significant evidence for changes in FAM processes and that combining olaparib with either an inhibitor of FAO or FAS further reduces tumor cell viability. The presence of DNA damage is thought to be able to alter metabolic pathways which in turn may regulate DNA repair processes [62]. Metabolic reprogramming is also thought to be involved in persister cell phenotypes [63]. Our data suggest it may be possible to target metabolic alterations to promote therapeutic efficacy. In support of this finding, a previous report demonstrated efficacy of combining PARP inhibition with an inhibitor of fatty acid synthase [64]. Utilization of metabolomic approaches may provide more comprehensive understanding of PARPi-induced metabolic rewiring and potential vulnerabilities which may be exploited to enhance treatment efficacy.

## 5. Conclusions

This study highlights the importance of detailed characterization of responses to treatment, which can inform both on therapeutic MOA and mechanisms of adaptation. This insight may lead to the development of strategies to enhance and/or prolong efficacy of treatment. We have identified ATM inhibition as a means to enhance PARPi utility and shown that targeting PARPi-induced EMT and metabolic alterations may be strategies to combat adaptation. More work is needed to better understand these strategies and investigate their translational potential. Though informative, this study is limited by use of in vitro models restricted to the LNCaP lineage, use of AbiR cells to validate C4-2B transcriptomic data rather than performing independent RNA-seq, and duration of treatment going only to 5 days. Future work will make use of additional models with increasing clinical relevance and incorporation of more powerful technologies such as single-cell RNA sequencing at later treatment timepoints to further characterize responses to treatment and adaptive mechanisms which may drive progression and treatment failure.

## Data Availability

The original contributions presented in this study are included in the article/Supplementary Material. Sequencing data and analysis are available as detailed in the “Section 2”. Further inquiries regarding raw data supporting this study can be directed to the corresponding author, and data are available upon request.

## Supplementary Materials

The following supporting information can be downloaded at: https://www.mdpi.com/article/10.3390/cancers18152381/s1.
