# Supplementary material for: Molecular Characterization of PARP Inhibitor Response Reveals Co-Targeting Strategies in Advanced Prostate Cancer
Source: Cancers (Basel). 2026 Jul 23;18(15):2381. doi: 10.3390/cancers18152381 (PMC13464659; doi:10.3390/cancers18152381)
Supplement: Supplementary file 1 [file cancers-18-02381-s001.zip › cancers-4391978-supplementary.pdf]

Molecular Characterization of PARP Inhibitor Response Reveals Co-Targeting Strategies in Advanced Prostate Cancer

Bryan Correa Gonzalez, Akshaya Karthikeyan, Love A. Moore, Anamitra Bhaumik, Ethan Sandoval , Marion Hardy, Ryan R. Davis, Neelu Batra, Christopher A. Lucchesi, Allen C. Gao, Hong Li, John D. McPherson, Marc Dall'Era and Alan P. Lombard

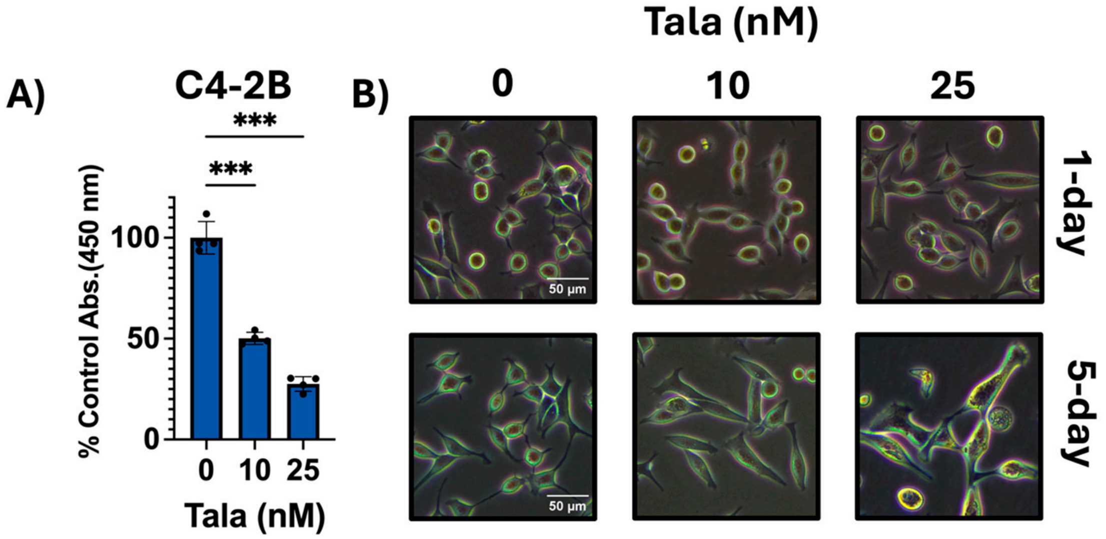

.

Supplemental Figure S1. Assessment of response to talazoparib in C4-2B cells. A. Cell viability assays show sensitivity of C4-2B cells to talazoparib treatment at 5-days via CCK-8. Data is presented as a % of control viability +/- standard deviation. Data were analyzed using ordinary one-way ANOVA with Dunnett's multiple comparison test (n = 4 replicates/condition). **B.** Phase contrast microscopy reveals morphology of treated C4-2B cells with indicated doses of talazoparib at indicated timepoints. Scale bars represent 50μm and apply to all panels. All images were acquired using identical magnification. All data is representative of 3 independent experiments. *** = p-value ≤ 0.001. Tala = talazoparib.

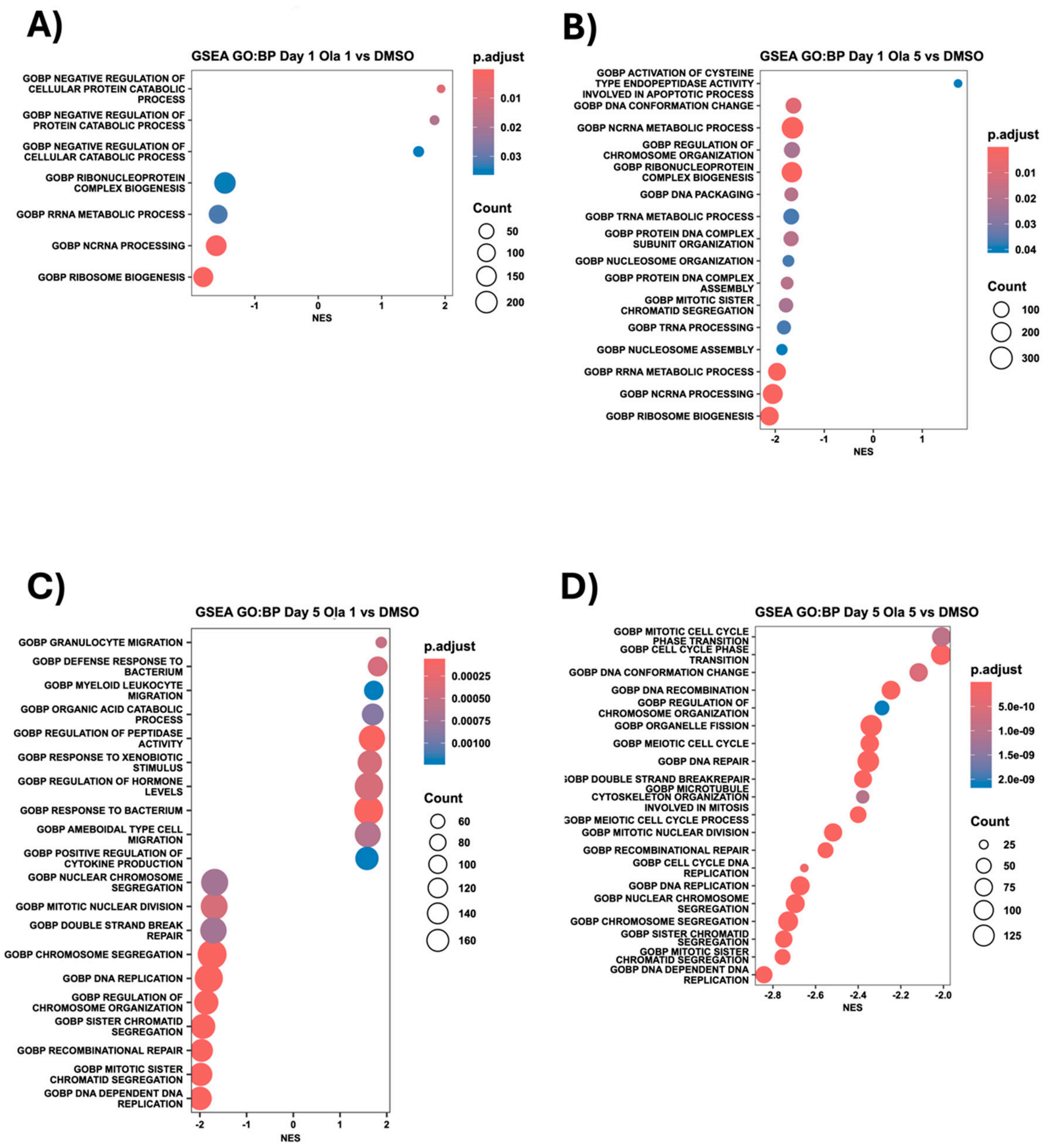

Supplemental Figure S2: Gene set enrichment analysis displaying Gene Ontology: Biological Process (GO:BP) gene sets enriched in C4-2B cells treated with 1 μM or 5 μM olaparib for 1 or 5 days. GSEA dot plots displaying top enriched GO:BP pathways comparing olaparib (Ola) treated cells to respective vehicle (DMSO) controls. Results are displayed for **A.** 1-day, Ola 1 μM. **B.** 1-day, Ola 5 μM. **C.** 5-day, Ola 1 μM. **D.** 5-day, Ola 5 μM. Up to top 20 most significant pathways by padj are displayed for each experimental condition.

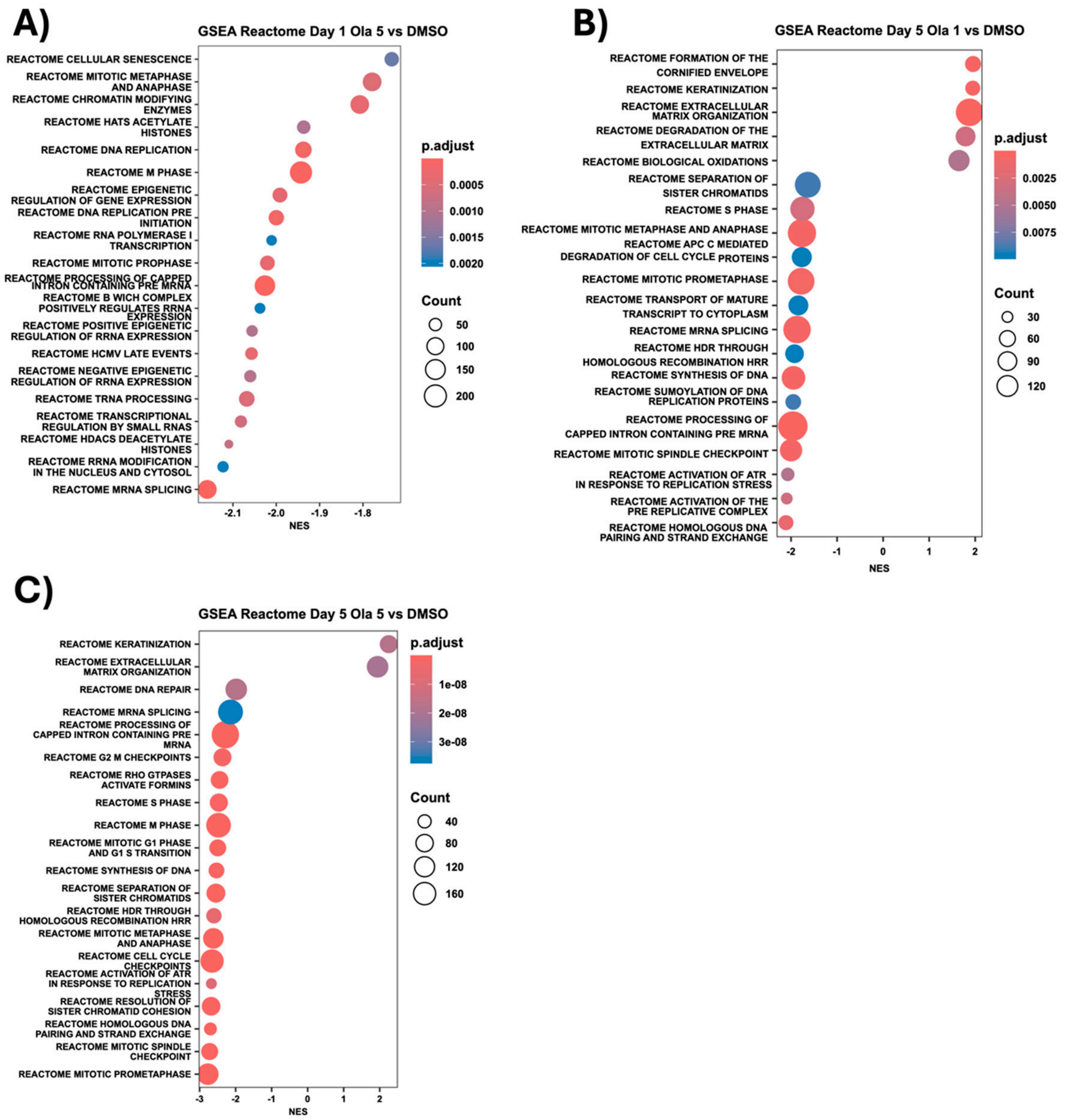

Supplemental Figure S3: Gene set enrichment analysis displaying Reactome Pathway gene sets enriched in C4-2B cells treated with 1 µM or 5 µM olaparib for 1 or 5 days. GSEA dot plots displaying top enriched Reactome pathways comparing olaparib (Ola) treated cells to respective vehicle (DMSO) controls. Results are displayed for **A.** 1-day, Ola 5 µM. **B.** 5-day, Ola 1 µM. **C.** 5-day, Ola 5 µM. Up to top 20 most significant pathways by padj are displayed for each experimental condition.

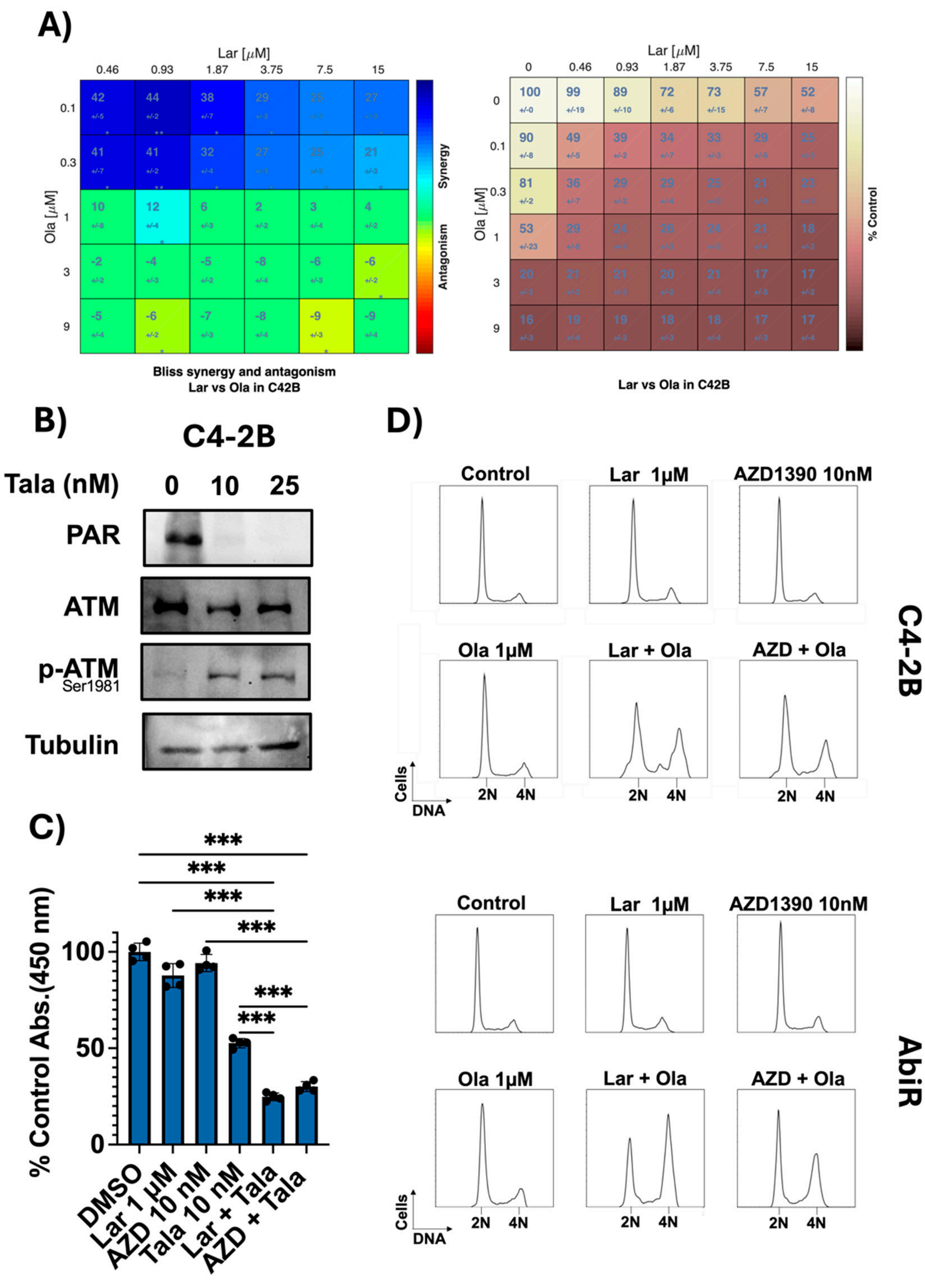

Supplemental Figure S4: ATM inhibition significantly enhances PARPi response in CRPC cells. A. Synergy matrix (left) and dose-response matrix (right) obtained from COMBENEFIT demonstrates synergy results between olaparib (Ola) and lartesertib (Lar) using Bliss model. Standard deviation is presented as +/-. Data is representative of 3 independent experiments where each experiment had 3 technical replicates. **B.** Western blots reveal expression of indicated proteins in response to 1-day talazoparib treatment at the indicated dosages in C4-2B cells. Tubulin served as a loading control. **C.** Cell viability assays show response of C4-2B cells to indicated 5-day treatments via CCK-8. Data is presented as a % of control viability +/- standard deviation. Data were analyzed using ordinary one-way ANOVA with Sidak's multiple comparison test (n = 4 replicates/condition). **D.** Representative DNA content histograms of cell cycle distribution via flow

cytometry of C4-2B (top) and AbiR (bottom) cells following 5 days of treatment. DNA content is measured by propidium iodide intensity. 2N peak corresponds to cells in the G0/G1 cell cycle phase while 4N peak corresponds to cells in G2/M phase. Combination treatment of ATM inhibitors with olaparib induce a significant accumulation of cells in G2/M phase. * = p-value ≤ 0.05, ** = p-value ≤ 0.01, *** = p-value ≤ 0.001, ns = not significant. Lar = lartesertib. AZD = AZD1390. Ola = olaparib. Tala = talazoparib. All data is representative of 3 independent experiments.

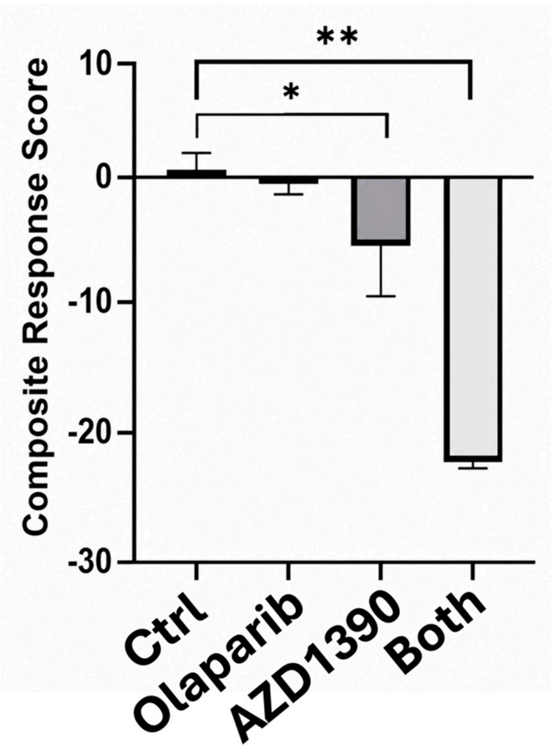

Supplemental Figure S5: Assessment of PARPi and ATMi combination in patient-derived prostate tumoroid model. Composite scores (described in Materials and Methods) reveal response to olaparib (10µM), AZD1390 (10nM), or a combination in a patient derived prostate tumoroid model. Significance was assessed via 2-way Anova. * = p-value ≤ 0.05, ** = p=value ≤ 0.005.

Supplementary Table S1: RIN values for RNA-sequencing samples

Sample ID	RIN
C4-2B Ola 0µM-1, Day 1	9.2
C4-2B Ola 0µM-2, Day 1	9.4
C4-2B Ola 0µM-3, Day 1	9.2
C4-2B Ola 1µM-1, Day 1	9.5
C4-2B Ola 1µM-2, Day 1	9.4
C4-2B Ola 1µM-3, Day 1	9.3
C4-2B Ola 5µM-1, Day 1	9.4
C4-2B Ola 5µM-2, Day 1	9.5
C4-2B Ola 5µM-3, Day 1	9.3
C4-2B Ola 0µM-1, Day 5	9.4
C4-2B Ola 0µM-2, Day 5	9
C4-2B Ola 0µM-3, Day 5	9.2
C4-2B Ola 1µM-1, Day 5	9.3
C4-2B Ola 1µM-2, Day 5	9.2
C4-2B Ola 1µM-3, Day 5	9.2
C4-2B Ola 5µM-1, Day 5	9.1
C4-2B Ola 5µM-2, Day 5	9.1
C4-2B Ola 5µM-3, Day 5	9.2
